# Characterization of Bicistronic Transcription in Budding Yeast

**DOI:** 10.1128/mSystems.01002-20

**Published:** 2021-02-23

**Authors:** Baojun Wu, Murray P. Cox

**Affiliations:** a Statistics and Bioinformatics Group, School of Fundamental Sciences, Massey University, Palmerston North, New Zealand; b Bio-Protection Research Centre, Massey University, Palmerston North, New Zealand; Pacific Northwest National Laboratory

**Keywords:** yeast, bicistronic transcripts, modulation of translation, *trans*-splice mechanisms

## Abstract

Bicistronic transcripts (operon-like transcripts) have occasionally been reported in eukaryotes, including unicellular yeasts, plants, and humans, despite the fact that they lack *trans*-splice mechanisms. However, the characteristics of eukaryotic bicistronic transcripts are poorly understood, except for those in nematodes. Here, we describe the genomic, transcriptomic, and ribosome profiling features of bicistronic transcripts in unicellular yeasts. By comparing the expression level of bicistronic transcripts with their monocistronic equivalents, we identify two main categories of bicistronic transcripts: highly and lowly expressed. These two categories exhibit quite different features. First, highly expressed bicistronic transcripts have higher conservation within and between strains and shorter intergenic spacers with higher GC content and less stable secondary structure. Second, genes in highly expressed bicistronic transcripts have lower translation efficiency, with the second gene showing statistically significant lower translation efficiency than the first. Finally, the genes found in these highly expressed bicistronic transcripts tend to be younger, with more recent origins. Together, these results suggest that bicistronic transcripts in yeast are heterogeneous. We further propose that at least some highly expressed bicistronic transcripts appear to play a role in modulating monocistronic translation.

**IMPORTANCE** Operons, where a single mRNA transcript encodes multiple adjacent proteins, are a widespread feature of bacteria and archaea. In contrast, the genes of eukaryotes are generally considered monocistronic. However, a number of studies have revealed the presence of bicistronic transcripts in eukaryotes, including humans. The basic features of these transcripts are largely unknown in eukaryotes, especially in organisms lacking *trans*-splice mechanisms. Our analyses characterize bicistronic transcripts in one such eukaryotic group, yeasts. We show that highly expressed bicistronic transcripts have unusual features compared to lowly expressed bicistronic transcripts, with several features influencing translational modulation.

## INTRODUCTION

The organization of multiple adjacent genes into a single polycistronic transcript is commonplace in bacteria and archaea (1). In contrast, polycistronic transcription was once thought to be absent from or exceedingly rare in eukaryotic nuclear genomes (2, 3). However, bicistronic transcripts are now known to be common in nematodes (3–6). While in prokaryotes the translation of multiple proteins from a polycistronic transcript occurs through multiple independent translation initiations, polycistronic transcripts in eukaryotes first must be processed into individual mRNAs before being translated in the cytoplasm (7). Consequently, the downstream genes in a eukaryotic polycistronic transcript lack the cap structure necessary for mRNA translation. Nematodes can circumvent this problem through *trans*-splicing of a short “spliced leader” RNA onto the 5′ end of the downstream genes that provides the cap structure needed for translation (8).

However, bicistronic transcripts have also been observed in other eukaryotes beyond nematodes. Ever more studies suggest that bicistronic transcripts are present in yeasts (9–11), plants (12, 13), and humans (14), although their distribution is sporadic, and it does not seem likely that they represent an ancestral eukaryotic trait (15). One striking point is that these organisms, in contrast to nematodes, lack a *trans*-splicing mechanism (3). Thus, these cryptic transcripts may not be effectively translated into peptides, and it is unclear whether these bicistronic transcripts are simply molecular errors. Previous transcriptome studies in the model organism Saccharomyces cerevisiae have shown the widespread presence of bicistronic transcripts in various strains and experimental conditions but often as little more than a side note (9, 10). Here, to better understand their genomic, transcriptomic, and ribosome profiling features, we systematically characterize bicistronic transcripts in unicellular yeast.

## RESULTS

### Bicistronic transcripts outnumber multicistronic transcripts.

In budding yeast Saccharomyces cerevisiae strain SLS045 (a S288C background strain), we identified 380 transcripts encoding two or more genes with unidirectional orientation (operon-like transcripts) that are supported by at least two transcript isoform sequencing (TIF-seq) reads. Only 13 transcripts (3%) contain more than two genes (all are tricistronic transcripts), with the other 367 transcripts containing only two genes (see Table S1 in the supplemental material). This suggests that operon-like structures are consistently smaller than those in prokaryotic systems. One caveat is that very long transcripts are relatively difficult to obtain; therefore, transcripts encoding more than three genes may be missed. Eight out of the 13 tricistronic transcripts coexist with bicistronic variants, suggesting that the structure of these operon-like transcripts is flexible. The functional annotation of genes in tricistronic transcripts shows that these genes are enriched with ribosomal subunits but does not support the notion that they are necessarily functional clusters (Table S1). Due to the rarity of tricistronic transcripts, we focus on the more common form, bicistronic transcripts, in the remainder of this study.

10.1128/mSystems.01002-20.4 TABLE S1 List of all bicistronic transcripts and multicistronic transcripts in Saccharomyces cerevisiae SLS045. Download Table S1, XLSX file, 0.04 MB. Copyright © 2021 Wu and Cox. 2021 Wu and Cox. https://creativecommons.org/licenses/by/4.0/ This content is distributed under the terms of the Creative Commons Attribution 4.0 International license.

### Bicistronic transcripts can be expressed more highly than their monocistronic transcripts.

The possibility exists that bicistronic transcripts, like many other processes that can generate transcriptome diversity, may primarily be molecular errors. Several studies have indicated that molecular errors across various organisms are less common in highly expressed genes (16–19). Therefore, the rate of bicistronic transcripts should be expected to decrease relative to their monocistronic gene expression level if bicistronic transcripts largely arise from molecular errors.

**FIG 1:**
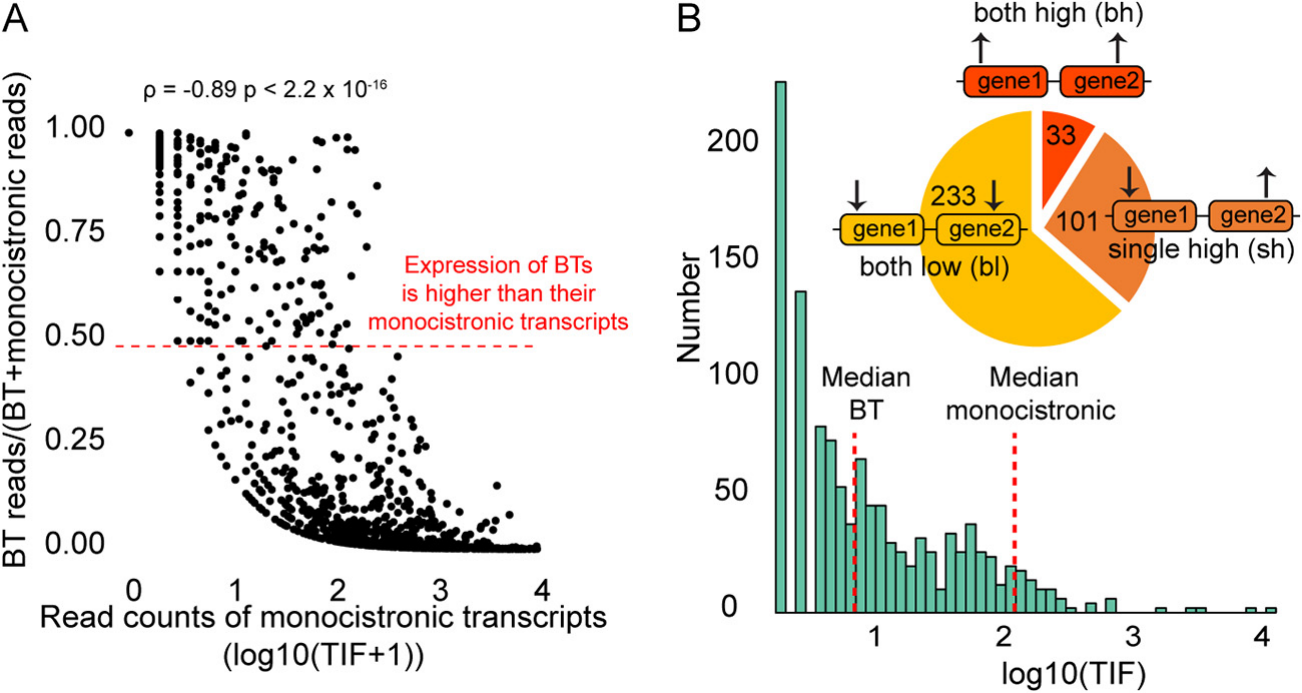
Expression patterns of bicistronic transcripts in Saccharomyces cerevisiae. (A) The rate of bicistronic transcript (BT) expression decreases with monocistronic transcript expression level. The 0.50 rate indicates equal expression levels between bicistronic transcripts and their monocistronic transcripts and is shown by a dotted red line. (B) Expression distribution of bicistronic transcripts. Median values of bicistronic transcripts and monocistronic transcripts are labeled in the histogram. The pie chart represents the proportion of the three categories of bicistronic transcripts: both high (bh), single high (sh), and both low (bl). Numbers in the pie chart refer to the number of transcripts in each of these classes.

Consistent with this, we find that the rate of bicistronic transcripts is negatively correlated with their monocistronic expression level (Spearman’s ρ = −0.89, *P* < 2.2 × 10^−16^; Fig. 1A), suggesting that most bicistronic transcripts are molecular errors. However, a sizeable proportion of bicistronic transcripts are expressed more highly than their monocistronic transcripts (dots above red dashed line in Fig. 1A). Here, these bicistronic transcripts will be referred to as “highly expressed bicistronic transcripts.” This class is determined by the ratio between bicistronic and monocistronic transcript levels, not the expression level of bicistronic transcripts alone. We quantify the proportions of bicistronic transcripts and find that up to 35% of bicistronic transcripts are highly expressed (Fig. 1B and Table S1). These highly expressed bicistronic transcripts can be further subdivided into both high (bh) and single high (sh) categories: (i) the bh category is where both genes in a bicistronic transcript have higher expression than their monocistronic transcripts, and (ii) the sh category is where only one gene in a bicistronic transcript has higher expression than either monocistronic transcript. All remaining bicistronic transcripts are placed in the both low (bl) category, where the expression level of both genes in a bicistronic transcript is lower than those of either of their monocistronic transcripts. If highly expressed bicistronic transcripts were deleterious, we would not expect that they would be expressed more highly than their monocistronic transcripts. Thus, we speculate that bicistronic transcripts in the bl category are mostly molecular errors, whereas highly expressed bicistronic transcripts in the bh and sh categories may be functional. In the sections below, we characterize the genomic and transcriptomic properties of these three groups.

### Highly expressed rather than lowly expressed bicistronic transcripts can repress translation.

We propose above that highly expressed bicistronic transcripts are functional. Thus, we collected the small number of cases in which the function of bicistronic transcripts has been experimentally verified (Table 1). All five of these bicistronic transcripts fall within the highly expressed categories (bh and sh), and their absolute expression level varies greatly, ranging from 5 to 3,735 TIF reads under yeast extract-peptone-dextrose (YPD) growth conditions (Table 1). This indicates that the absolute expression level of bicistronic transcripts alone is not a useful measure of whether bicistronic transcripts are functional or not.

**TABLE 1:** Verified functions of bicistronic transcripts

Bicistronic transcript	Class	TIF^*a*^	Function
PMP1_YCR024C-B	bh	3735	YCR024C-B has a 3′ untranslated region that directs PMP1 subcellular localization (52)
YOR302W_CPA1	bh	1827	Bicistronic transcript negatively regulates translation of CPA1 (21)
RTC4_GIS2	sh	161	Bicistronic transcript negatively regulates translation of both monocistronic transcripts (22)
YMR147W_OSW5	bh	64	Ldo45 is the product of a splicing event that connects two adjacent genes and acts as key determinant of lipid droplet identity (24)
YNR068C_BSC5	sh	5	Bul3p is the product of a bypass event connecting two adjacent genes and acts as a negative regulator of Rsp5p-dependent ubiquitination (23)

a Expression level as measured in YPD growth conditions.

**FIG 2:**
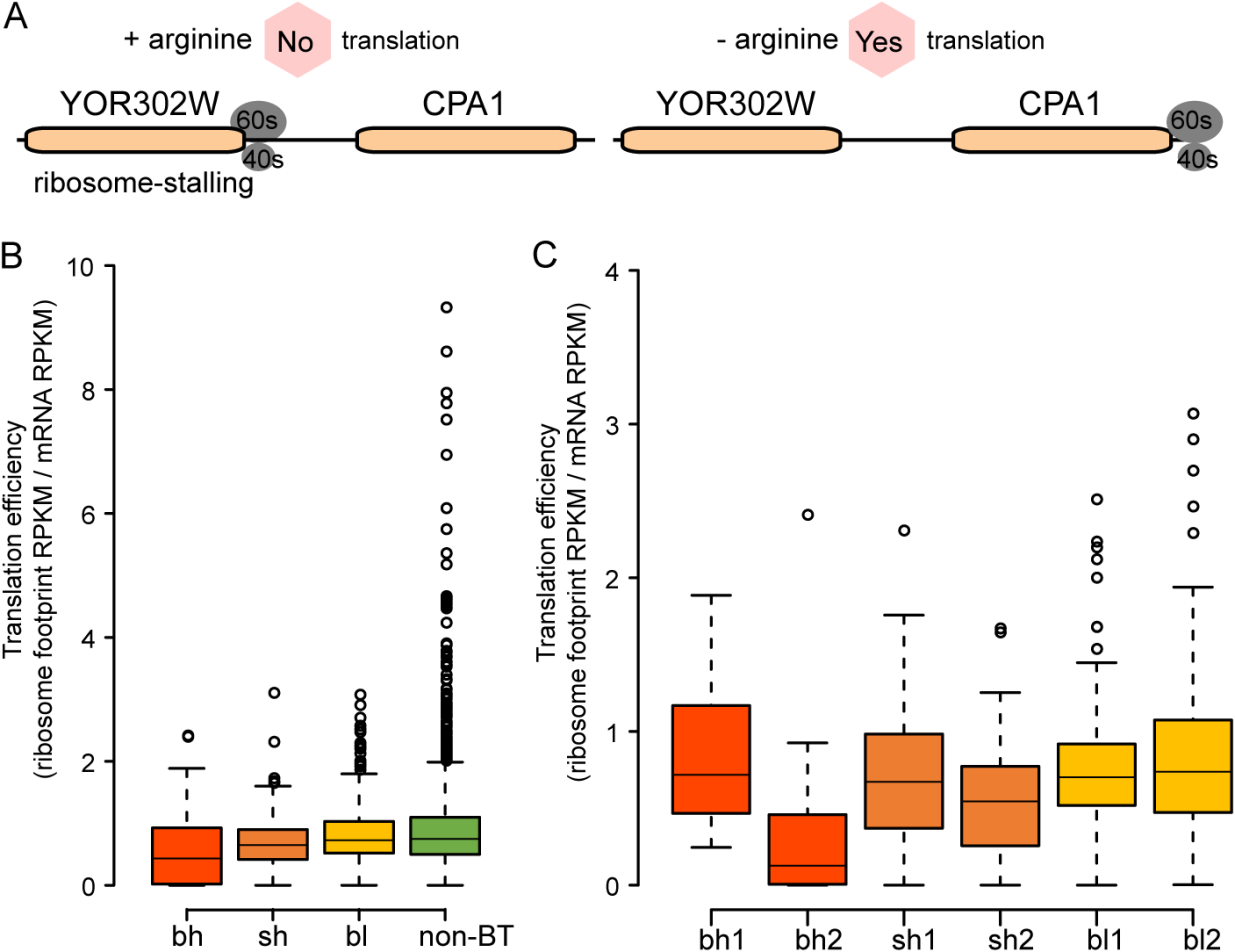
Translation efficiency of genes in bicistronic transcripts (BT). (A) Bicistronic transcript-mediated translational repression of CPA1. (B) Comparison of translation efficiency between bicistronic transcripts (bh, sh, and bl categories) and non-BT control genes. Except for the bl/non-BT pair, all pairs are significantly different (*P* < 0.05). (C) Comparison of translation efficiency between the first and second gene in bh, sh, and bl bicistronic transcripts. The number 1 indicates the first gene, and 2 indicates the second gene in the bicistronic transcripts. bh1/bh2 and sh1/sh2 pairs are significantly different (*P* < 0.05). Statistical significance was determined with the two-sided Mann-Whitney U test.

Among these five cases (20–24), YOR302W_CPA1 is the most well characterized. Bicistronic transcription together with translation of YOR302W can repress translation of CPA1 transcripts via ribosome stalling when arginine is present (Fig. 2A) (21, 25, 26). RTC4_GIS2 is another case where bicistronic transcription regulates translation of monocistronic transcripts, although no clear mechanism is known for this phenomenon. In contrast, the bicistronic transcript RTC4_GIS2 can repress translation of both monocistronic transcripts (22).

To test whether the translation of genes in other bicistronic transcripts is repressed, we examine the translation efficiency (TE; ribosome footprint/mRNA) of genes belonging to bicistronic transcripts versus a genomic control of non-BT gene pairs (two adjacent genes with matched transcriptional direction but no evidence of bicistronic transcripts). The translation efficiency of genes in highly expressed bicistronic transcripts is significantly lower than that of non-BT genes, whereas genes in the lowly expressed category are not affected (Fig. 2B). To further figure out which gene is being repressed in the bicistronic transcript, the TE of the first gene (gene 1) and the second gene (gene 2) were compared. The first gene in the bicistronic transcripts is not affected in all three categories, but the second gene is repressed in the bh and sh categories (Fig. 2C). From this, we infer that the repressive role of bicistronic transcripts is more similar to the YOR302W_CPA1 case than RTC4_GIS2 (Table 1). Consistent with this, we find that the first gene tends to be more highly expressed in bicistronic transcripts than its equivalent monocistronic transcript (Fig. S2).

### Highly expressed bicistronic transcripts are more conserved than lowly expressed bicistronic transcripts both within and between strains.

**FIG 3:**
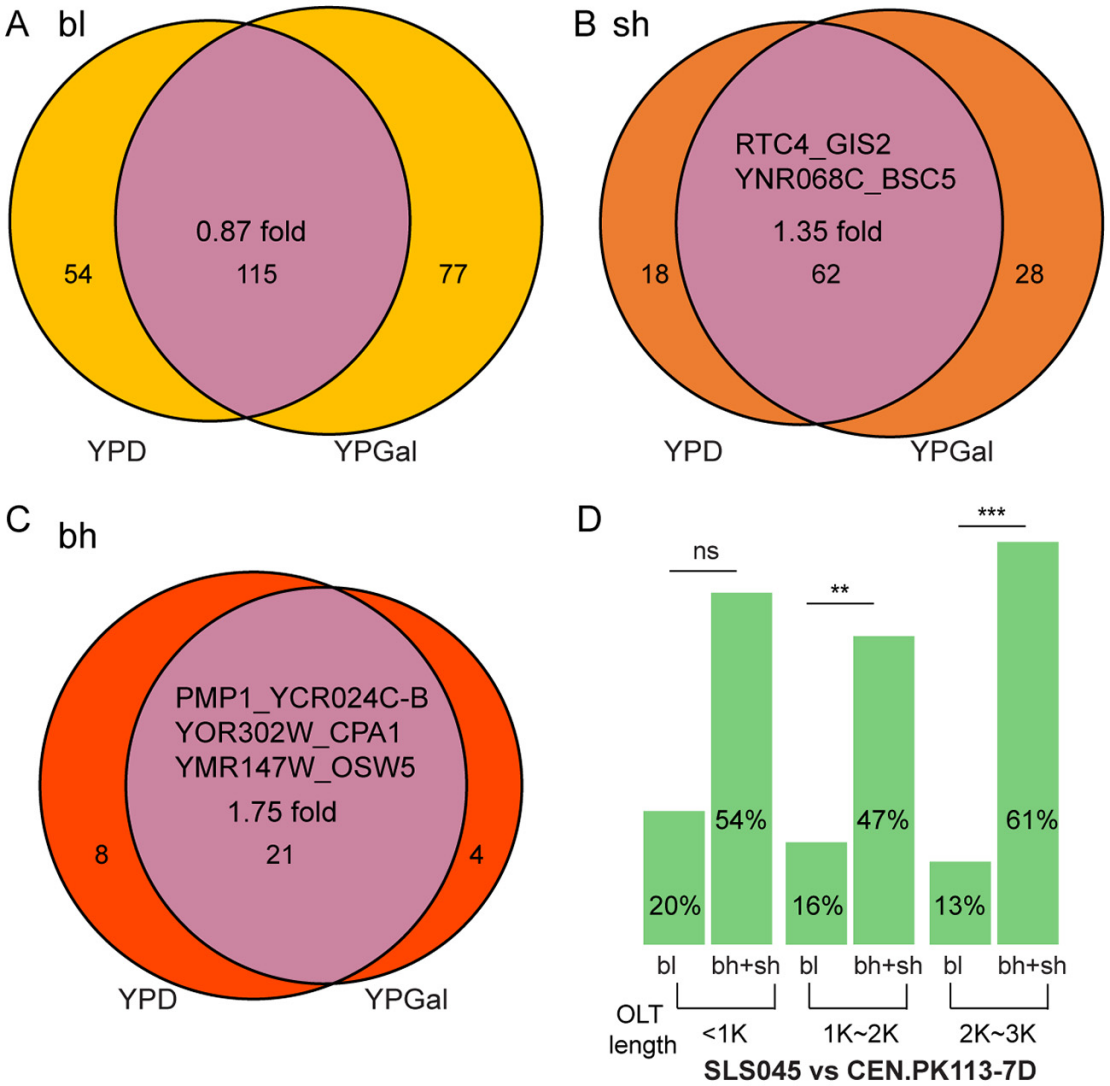
Conservation of bicistronic transcripts within and between strains. (A to C) Distribution of bicistronic transcripts under YPD and YPGal growth conditions. Functionally verified bicistronic transcripts are noted within the Venn diagrams. The fold change was calculated by the ratio of shared BTs/unique BTs in each category. (D) Shared proportion of three categories of bicistronic transcripts between two strains. The bicistronic transcript length includes the length of both genes and intergenic region. Statistical significance was determined with a two-sided Fisher test.

If highly expressed bicistronic transcripts are functional but lowly expressed bicistronic transcripts are molecular noise, we might expect highly expressed bicistronic transcripts to be more conserved than lowly expressed bicistronic transcripts. To test whether highly expressed bicistronic transcripts are more conserved, we first compared them in cells grown under two conditions: yeast extract-peptone-dextrose (YPD) and yeast extract-peptone-galactose (YPGal) growth conditions (10). We identified 278 and 307 bicistronic transcripts in YPD and YPGal (Table S1), respectively. The proportion of shared bicistronic transcripts is larger than that of unique bicistronic transcripts in the highly expressed categories (bh and sh) but not in the lowly expressed bl category (Fig. 3A to C). Moreover, all functionally verified cases (Table 1) are in the highly expressed categories (Fig. 3A to C).

We then searched for bicistronic transcripts in S. cerevisiae CEN.PK113-7D grown in YPD using MinION direct transcriptome sequencing (RNA-seq) reads (27), and we calculated how many bicistronic transcripts in the SLS045 strain are found in strain CEN.PK113-7D. Comparison between the two strains reveals that highly expressed bicistronic transcripts are more conserved than lowly expressed bicistronic transcripts (Fig. 3D). These results further support the proposition that highly expressed bicistronic transcripts are more likely functional.

### Structural characteristics of intergenic regions vary between highly and lowly expressed bicistronic transcripts.

**FIG 4:**
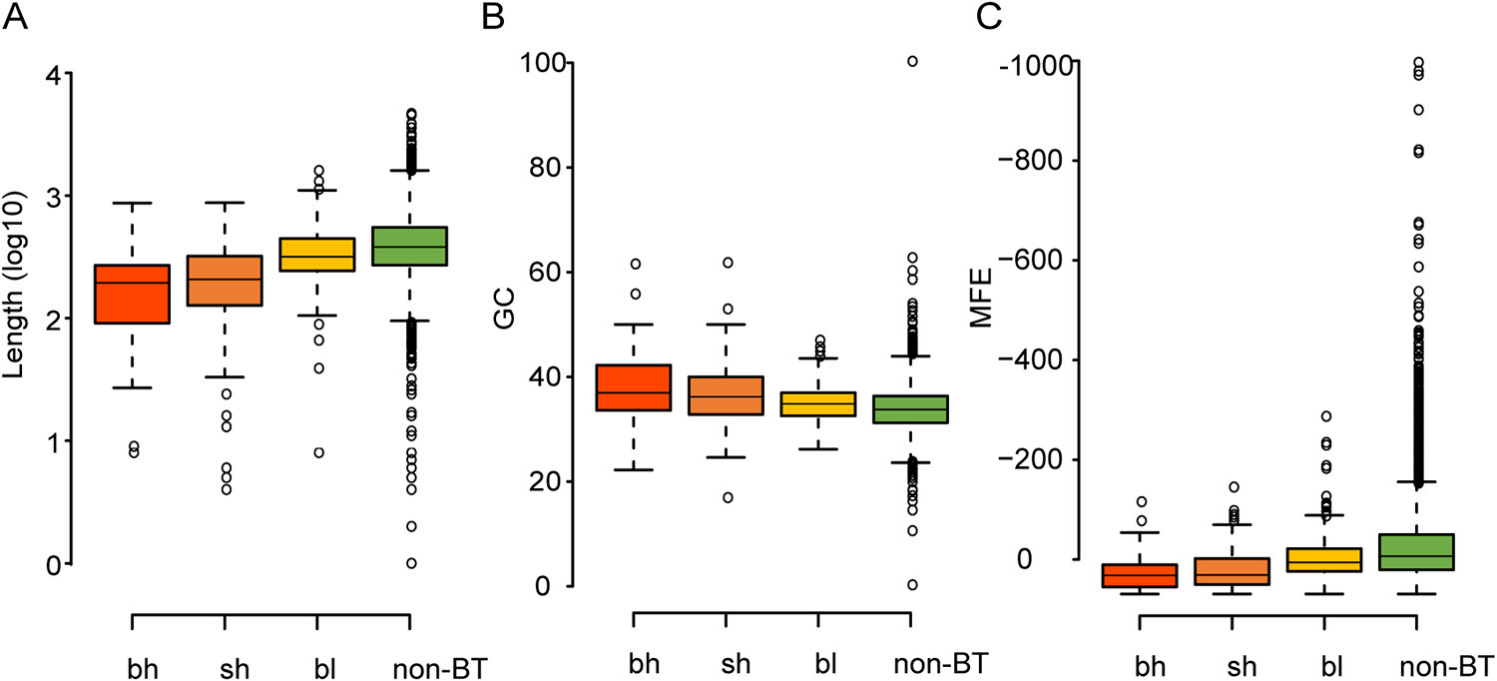
Characteristics of intergenic regions of bicistronic transcripts (BT) in Saccharomyces cerevisiae. (A to C) Comparison of intergenic distance (A), intergenic GC content (B), and minimum folding energy (MFE) of intergenic regions (C) between bh, sh, and bl bicistronic transcripts and control non-BT gene pairs. All pairs are significantly different (*P* < 0.05), except the bh/sh pair. In panel C, more negative minimum folding energy indicates more stable secondary structure. Statistical significance was determined with the two-sided Mann-Whitney U test.

To identify a possible basis for these patterns, we explored the structural characteristics of intergenic regions. When comparing intergenic spacer sequences in control non-BT pairs, the intergenic spaces of bicistronic transcript pairs are generally shorter and have a higher GC content and less stable secondary structure (Fig. 4A to C). These features are predicted to contribute to noncoding readthrough (28). For instance, the intergenic regions in bicistronic transcripts form much less stable secondary structures, which may facilitate the RNA polymerase to pass through the intergenic region without detaching. Similarly, high GC content and short length are also characteristics of intergenic regions within bacterial operons compared with nonoperon intergenic regions (29, 30). Thus, it has been proposed that these factors are a common mechanism to facilitate readthrough transcription. Moreover, we compare these intercistronic characteristics among three categories of bicistronic transcripts. The highly expressed bicistronic transcripts (bh and sh) tend to have shorter spacers, higher GC content, and less stable secondary structures than lowly expressed bicistronic transcripts (bl) (Fig. 4A to C), again suggesting that genes in the bl class are dominated by nonfunctional noisy transcription.

We investigated sequence conservation in the intergenic regions of bicistronic transcripts using interspecific comparisons between S. cerevisiae and S. paradoxus (Fig. S1). In contrast to structural features, sequence conservation is not different between the categories, which suggests that sequence conservation of intergenic regions in bicistronic transcripts is not needed for readthrough.

10.1128/mSystems.01002-20.1 FIG S1 Genetic distance in intergenic regions of bicistronic transcripts (BT). Comparison of genetic distances was performed between the bh, sh, and bl classes of bicistronic transcripts and control non-BT pairs (two adjacent genes with matched transcriptional direction but no evidence of bicistronic transcripts). Genetic distance was calculated using orthologs between Saccharomyces cerevisiae and Saccharomyces paradoxus CBS432. Statistical significance was determined with a two-sided Mann-Whitney U test. Download FIG S1, PDF file, 0.3 MB. Copyright © 2021 Wu and Cox. 2021 Wu and Cox. https://creativecommons.org/licenses/by/4.0/ This content is distributed under the terms of the Creative Commons Attribution 4.0 International license.

10.1128/mSystems.01002-20.2 FIG S2 Proportion of cases in which bicistronic expression is larger than monocistronic expression for gene 1 and gene 2. Download FIG S2, PDF file, 0.7 MB. Copyright © 2021 Wu and Cox. 2021 Wu and Cox. https://creativecommons.org/licenses/by/4.0/ This content is distributed under the terms of the Creative Commons Attribution 4.0 International license.

### The origin of highly expressed bicistronic transcripts is relatively young.

**FIG 5:**
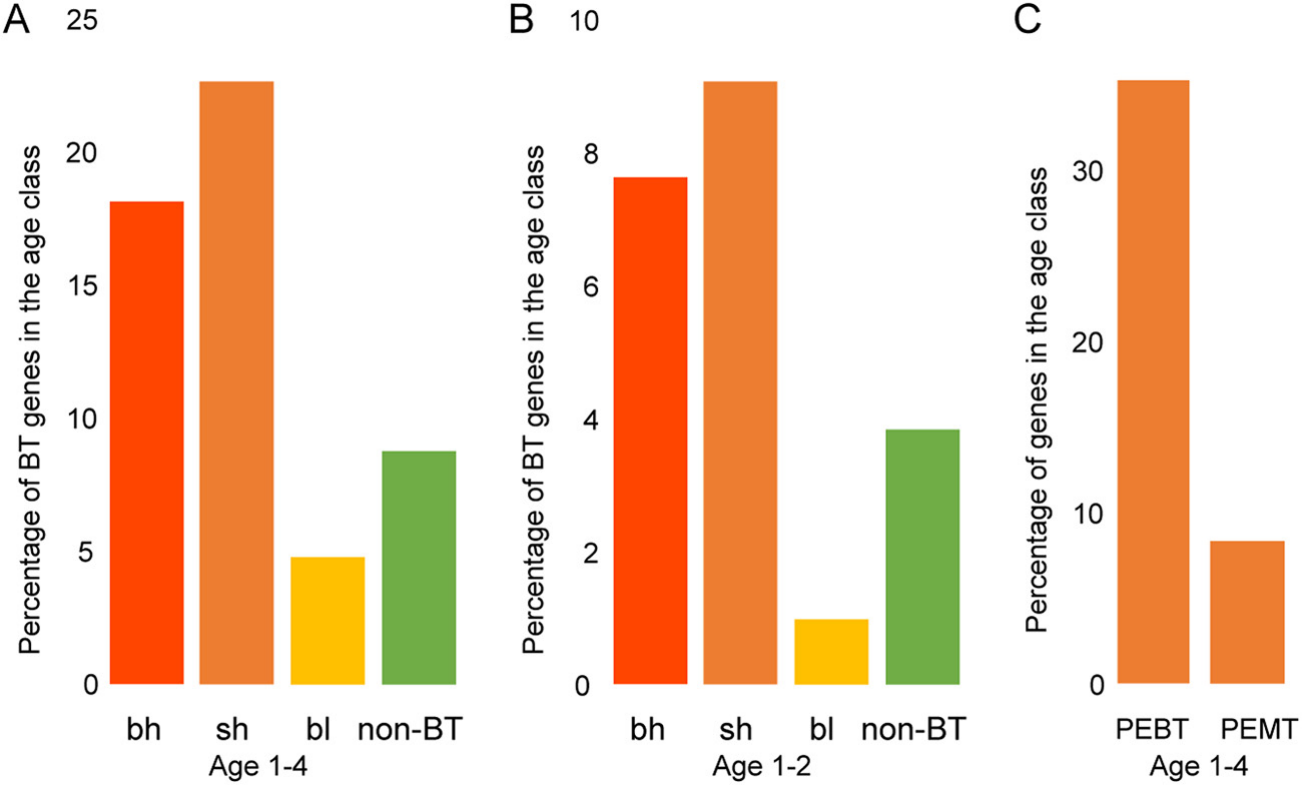
Comparison of gene ages between bicistronic gene classes (bh, sh, and bl) and control non-BT genes. The young age refers to conservation level 1 to 4 out of a total of 10 levels. (A) Ages 1 to 4. (B) Ages 1 to 2. Statistical significance was determined with a two-sided Fisher test. In panel A, all pairs are significantly different (*P* < 0.05), except the bh/sh pair. In panel B, all pairs are significantly different (*P* < 0.05), except the bh/sh and bh/non-BT pairs. (C) Percentage of genes in the age class between PEBT (preferentially expressed as a bicistronic transcript) and PEMT (preferentially expressed as a monocistronic transcript) groups. The difference is significant (*P* < 0.05).

Finally, we tracked down the origin of bicistronic transcripts. Carvunis et al. (31) assigned yeast genes into 10 age categories, and on the basis of gene ages, we find that highly expressed bicistronic transcripts are enriched for young genes compared to lowly expressed bicistronic transcripts and control non-BT pairs (Fig. 5A and B). This pattern suggests that the birth of highly expressed bicistronic transcripts is relatively recent.

Likely because they are young, the genes in highly expressed bicistronic transcripts have shorter lengths than lowly expressed bicistronic transcripts (Fig. S3). We explored the relationships between expression patterns (monocistronic/bicistronic transcripts) and age (old/young) in the sh class and found that genes preferentially expressed as a monocistronic transcript are older than those preferentially expressed as a bicistronic transcript (Fig. 5C). This pattern suggests that the emergence of young genes is important for the formation of bicistronic transcripts.

10.1128/mSystems.01002-20.3 FIG S3 Length of coding genes in bicistronic transcripts. Comparison was performed between the bh, sh and bl classes of bicistronic transcripts and control non-BT pairs (two adjacent genes with matched transcriptional direction but no evidence of bicistronic transcripts). Statistical significance was determined with a two-sided Mann-Whitney U test. Download FIG S3, PDF file, 0.2 MB. Copyright © 2021 Wu and Cox. 2021 Wu and Cox. https://creativecommons.org/licenses/by/4.0/ This content is distributed under the terms of the Creative Commons Attribution 4.0 International license.

Genes that emerged in recent evolutionary time are enriched for stress-responsive genes in many yeast species (32), which indicates that highly expressed bicistronic transcripts respond to stress, as in the case of the functionally validated gene pair YOR302W_CPA1. Highly expressed bicistronic transcripts have responses to various forms of stress, such as osmotic stress (Table 2). Functional enrichment finds that highly expressed bicistronic transcripts have overrepresented associations with 12 GO terms, particularly cell wall organization and sporulation (Table 3). In budding yeast, some adjacent gene pairs are nonrandomly clustered, which can result in tighter transcriptional coordination. GO analysis of these adjacent, but nonbicistronic, gene pairs also reveals enriched functions for cell wall organization and sporulation (33), suggesting that bicistronic transcripts for these functions add to the transcriptional coordination of such genes in budding yeast.

**TABLE 2:** Cases of bicistronic transcripts in response to stress

Gene	Position in BT	Function
YBL075C	Second	Response to oxidative stress/heat shock
YCR104W	Second	Response to stress, fungal type cell wall organization
YFL020C	Second	Response to stress, fungal type cell wall organization
YHR106W	Second	Response to oxidative stress
YKL056C	Second	Cellular response to oxidative stress
YOR009W	Second	Response to stress, fungal type cell wall organization
YPL152W	Second	Response to osmotic stress

**TABLE 3:** GO enrichment of highly expressed bicistronic transcripts

GO term	Description	Count
GO:0030435	Sporulation resulting in formation of a cellular spore	8
GO:0071555	Cell wall organization	7
GO:0030437	Ascospore formation	6
GO:0050790	Regulation of catalytic activity	6
GO:0006513	Protein monoubiquitination	5
GO:0000209	Protein polyubiquitination	5
GO:0034613	Cellular protein localization	3
GO:0000413	Protein peptidyl-prolyl isomerization	3
GO:0048278	Vesicle docking	3
GO:0006729	Tetrahydrobiopterin biosynthetic process	2
GO:0071025	RNA surveillance	2
GO:1901137	Carbohydrate derivative biosynthetic process	2

## DISCUSSION

Here, we systematically investigate the features of bicistronic transcripts in a unicellular eukaryotic microorganism. Although most bicistronic transcripts are consistent with being molecular errors, we infer that a substantial proportion have properties that are consistent with them potentially being functional. In particular, 35% of bicistronic transcripts are expressed more highly than their monocistronic transcripts.

It is worth noting that the expression ratio between bicistronic transcripts and their monocistronic transcripts is variable and likely regulated by mRNA decay (9). For instance, disruption of the mRNA decay enzymes YOL149W (DCP1) and YGL173C (XRN1) leads to a substantial increase in the amount of monocistronic transcription for YMR181C relative to its corresponding bicistronic transcripts (RGM1-YMR181C, an sh bicistronic transcript in this study) (9). Enzymes of the mRNA decay system can also reshape both Escherichia coli operons and Caenorhabditis elegans operon-like transcripts (34, 35). This supports the proposition that mRNA decay is a widespread mechanism for modulating the expression patterns of bicistronic transcripts.

Extensive studies have suggested that upstream open reading frames (uORFs) often regulate translation of the main ORF (mORF) in various organisms (36–39). In these cases, the uORF-mORF transcript together with uORF translation often downregulates the translational efficiency of the mORF via ribosome stalling (40, 41). However, these repressive effects can be lifted when conditions change. Consequently, the regulatory role of uORFs is important for organisms to respond to changing environments (40).

Although different from traditional uORFs (which are typically just a few codons in length), the first gene in bicistronic transcripts can also be regarded as a uORF of the downstream gene. In yeast, a well-studied case is YOR302W_CPA1 (a bh bicistronic transcript in this study), where the upstream gene YOR302W mediates translation of downstream gene CPA1 (21). Cpa1 catalyzes a step in arginine biosynthesis and is only needed when arginine is deficient. When this is the case, leaky scanning of YOR302W (∼50% of ribosomes) can translate CPA1. However, when arginine is present, ribosomes become stalled during translation of YOR302W and prevent any ribosomes from reaching CPA1, reducing Cpa1 synthesis (42). In our study, we find that the translation efficiency of the second gene relative to the first gene in a bicistronic pair is significantly lower in highly expressed bicistronic transcripts (Fig. 2), strongly resembling the well-studied YOR302W_CPA1 case. Thus, we speculate that highly expressed bicistronic transcripts play a role in modulating translation patterns in yeast. In addition to modulating translation, bicistronic transcripts can also perform other roles in cell physiology, particularly under stress conditions. For instance, the merged Bul3 protein (YNR068C_BSC5, a sh bicistronic transcript in this study) inhibits Bul1/2p-independent endocytosis under poor-nitrogen-source conditions (Table 1) but is produced at only low levels under nonstress conditions (23).

Budding yeast have a significant incidence of functionally clustered coregulated gene pairings (33, 43, 44). Here, we find that gene pairs included in at least 7% of bicistronic transcripts (27 out of 367) are functionally related (see Table S2 in the supplemental material). For example, YBR092C (PHO3) is transcribed in a transcript together with YBR093C (PHO5), and both genes are involved in the same pathway of riboflavin metabolism according to KEGG. Studies have proposed bicistronic transcription as a mechanism contributing to coregulation of linked genes in nematodes (45). Therefore, we posit that bicistronic transcription partially contributes to the formation of tandem distribution of functional clusters in budding yeast.

10.1128/mSystems.01002-20.5 TABLE S2 Examples of genes in bicistronic transcripts with related functions. Download Table S2, XLSX file, 0.01 MB. Copyright © 2021 Wu and Cox. 2021 Wu and Cox. https://creativecommons.org/licenses/by/4.0/ This content is distributed under the terms of the Creative Commons Attribution 4.0 International license.

Saccharomyces cerevisiae has been widely used as a cell factory for the production of fuels, chemicals, pharmaceuticals, and food ingredients. Many metabolic clusters have been pioneered in this organism. Our findings may usefully inform synthetic biology in terms of how to effectively construct new metabolic operons in yeast. For instance, our results indicate that shorter spacers, higher GC content, and less stable intergenic secondary structure could increase polycistronic transcripts relative to their monocistronic transcripts (Fig. 4). Consequently, production of proteins from the cluster would be decreased. Thus, we propose that the design of synthetic metabolic operons should avoid these intergenic characteristics.

In conclusion, we have characterized bicistronic transcripts through analyses of genomic and transcriptomic/ribosome profiling. While the expression patterns of bicistronic transcripts generally match the expectations of the molecular error hypothesis, up to 35% of bicistronic transcripts have characteristics that make them strong candidates for functional entities. We suggest that highly expressed bicistronic transcripts modulate the translation of monocistronic transcripts as uORF-mORF pairs. Moreover, we provide a set of highly expressed bicistronic transcript candidates to facilitate targeted experiments. We anticipate that characterizing the functions of yeast bicistronic transcripts will be a key step to understanding bicistronic transcription in eukaryotic organisms that lack *trans*-splice mechanisms.

## MATERIALS AND METHODS

### Identification of bicistronic transcripts in S. cerevisiae SLS045.

The TIF-seq method can capture both the capped and polyadenylated ends of a transcript and is the gold standard for identifying polycistronic transcripts (10). The position and supported counts of transcript isoforms for S. cerevisiae SLS045 (an S288c background) are drawn from the study of Pelechano et al. (10). The latest annotation of S. cerevisiae S288C R64-2-1 was taken from the *Saccharomyces* Genome Database (SGD) (https://downloads.yeastgenome.org/sequence/S288C_reference/). The intersect function of bedtools v. 2.27 (46) was used to compare 5′ and 3′ ends of each transcript isoform against the gene position with −f 1.0 (100% coverage). If the transcript covers two genes, all genes within the transcript were assigned to one bicistronic transcript. Only bicistronic transcripts that were supported by at least two TIF reads are considered. Bicistronic transcripts found under two growth conditions (YPD and YPGal) are listed in Table S1.

### Identification of bicistronic transcripts in S. cerevisiae CEN.PK113-7D.

MinION direct RNA sequencing of S. cerevisiae CEN.PK113-7D grown in YPD was downloaded from NCBI BioProject no. PRJNA398797 (27). Seqtk v. 1.3 (https://github.com/lh3/seqtk) was used to convert fastq reads to fasta. Coding sequences of S288C R64-2-1 were downloaded from SGD (https://downloads.yeastgenome.org/sequence/S288C_reference/). Coding sequences were used as queries to BLASTn against each minION direct RNA read (minimum length >1 kb). We counted genes as a bicistronic transcript when genes were covered by a single mRNA read using >90% identity and >99% coverage, and only bicistronic transcripts that were supported by at least two mRNA reads are considered.

### Analyses of intergenic spacers and coding genes in bicistronic transcripts.

Intergenic regions of S. cerevisiae S288c R64-2-1 were downloaded from SGD (https://downloads.yeastgenome.org/sequence/S288C_reference/). The minimum folding energy (MFE) was computed using DAMBE v. 7 (47) with default parameters. The estimated age of yeast genes was taken from the study of Carvunis et al. (31). The mRNA reads per kilobase per million (RPKM) and corresponding footprint RPKM were taken from the study of Gerashchenko et al. (48). Translation efficiency was calculated using the ratio between ribosome footprint RPKM and mRNA RPKM. The genetic distance of intergenic regions in bicistronic transcripts was calculated using MEGA-CC with the Kimura 2-parameter model (49). SGD gene identifiers (IDs) were downloaded from the SGD database (50). The IDs of genes included in each highly expressed bicistronic transcript were submitted to DAVID v. 6.8 (51) to perform GO enrichment analysis, with a default EASE cutoff of 0.1. In addition, we manually checked the functional description of genes in highly expressed bicistronic transcripts using the SGD database (50).
