# Supplementary figures and images for: Characterization of Bicistronic Transcription in Budding Yeast
Source: mSystems. 2021 Feb 23;6(1):e01002-20. doi: 10.1128/mSystems.01002-20 (PMC8573964; doi:10.1128/mSystems.01002-20)

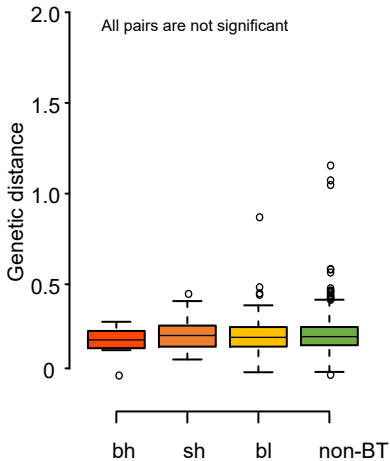

Supplement: FIG S1 [file msystems.01002-20-sf001.pdf]

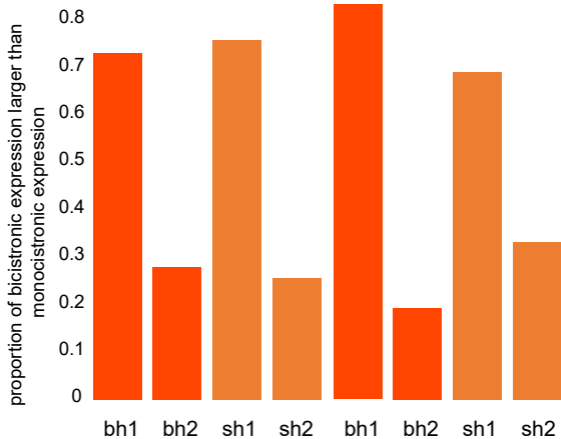

Supplement: FIG S2 [file msystems.01002-20-sf002.pdf]

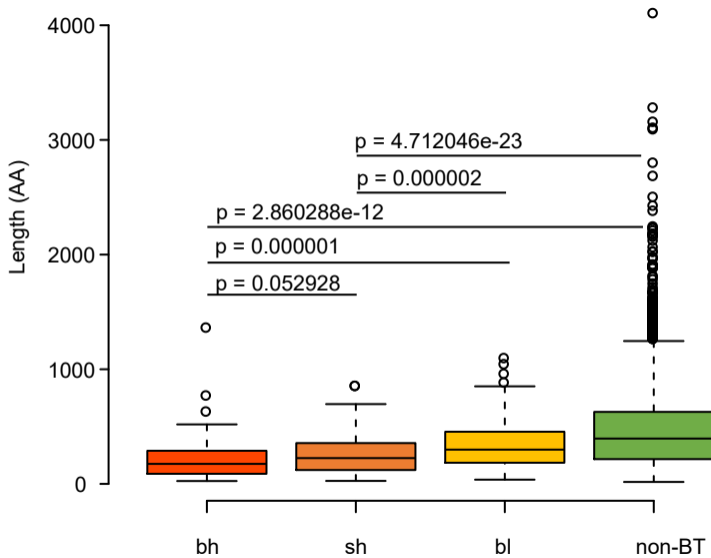

Supplement: FIG S3 [file msystems.01002-20-sf003.pdf]
